# Supplementary figures and images for: Metabolic Rewiring at the Pyruvate Node Drives Severe Pneumonia and T-Cell Suppression in Serotype 3 Streptococcus pneumoniae Infection
Source: bioRxiv. 2026 Feb 15:2026.02.13.705843. Preprint. [Version 1] doi: 10.64898/2026.02.13.705843 (PMC12918967; doi:10.64898/2026.02.13.705843)

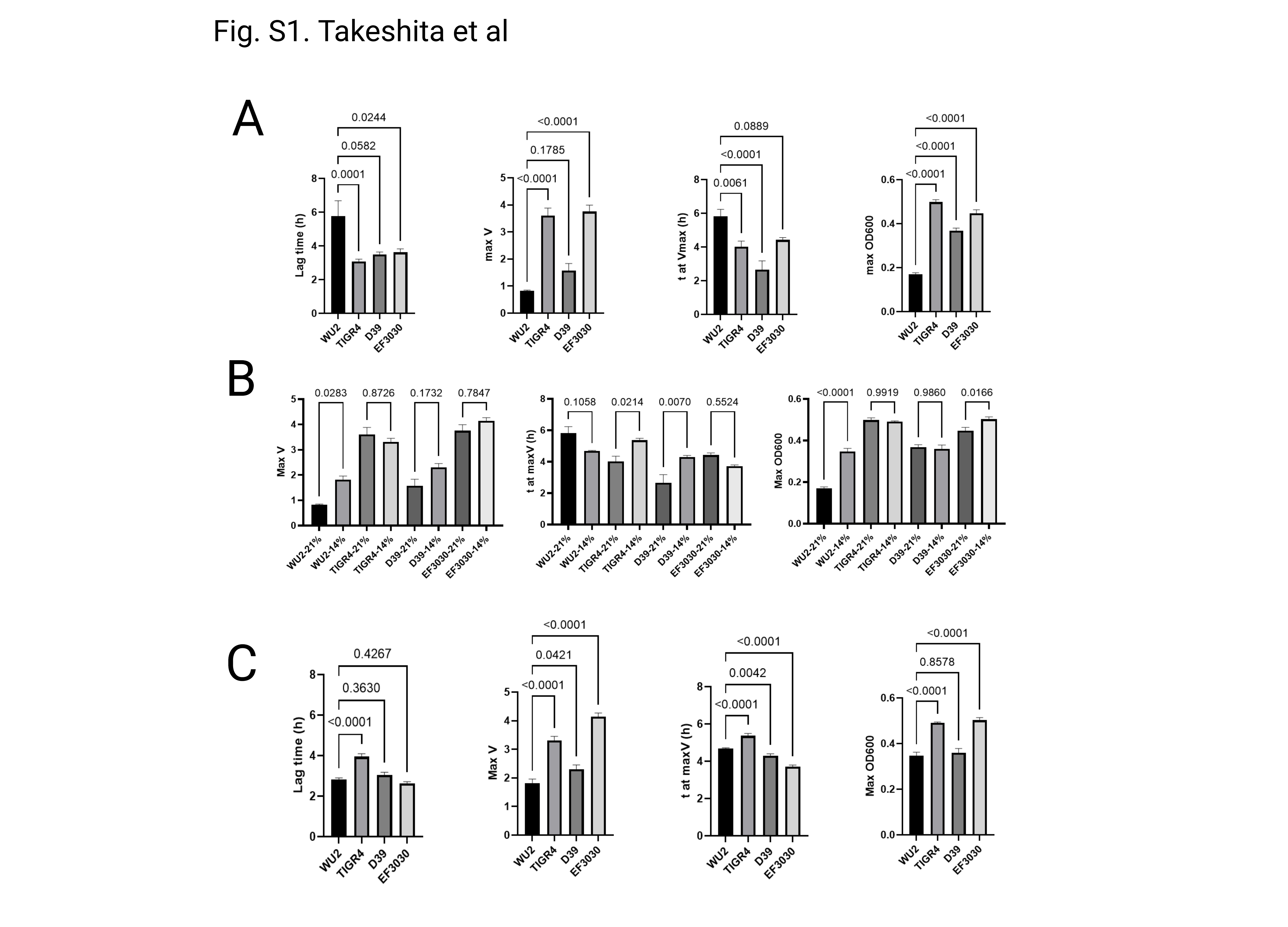

Supplement: Supplement 1 — Fig. S1. Growth kinetics of Streptococcus pneumoniae types under varying oxygen conditions. (A) Growth parameters of serotype 3 strain WU2 compared to TIGR4, D39, and EF3030 under nasopharyngeal conditions (21% O2) over 24 h. Bars represent mean values of lag time (h), maximum growth rate (Max V, 1/h), time to maximum growth rate (t at Max V, h), and maximum optical density (Max OD600). (B) Comparative analysis of WU2 growth parameters relative to TIGR4, D39, and EF3030 under lung conditions (14% O2) versus nasopharyngeal conditions, showing enhanced growth kinetics. (C) WU2 growth parameters under lung conditions (14% O2) compared to TIGR4, D39, and EF3030. Data in all panels are shown as mean ± SE. Statistical analyses in panels were performed using one-way ANOVA with Šidák’s multiple comparisons test. [file media-1.jpg]

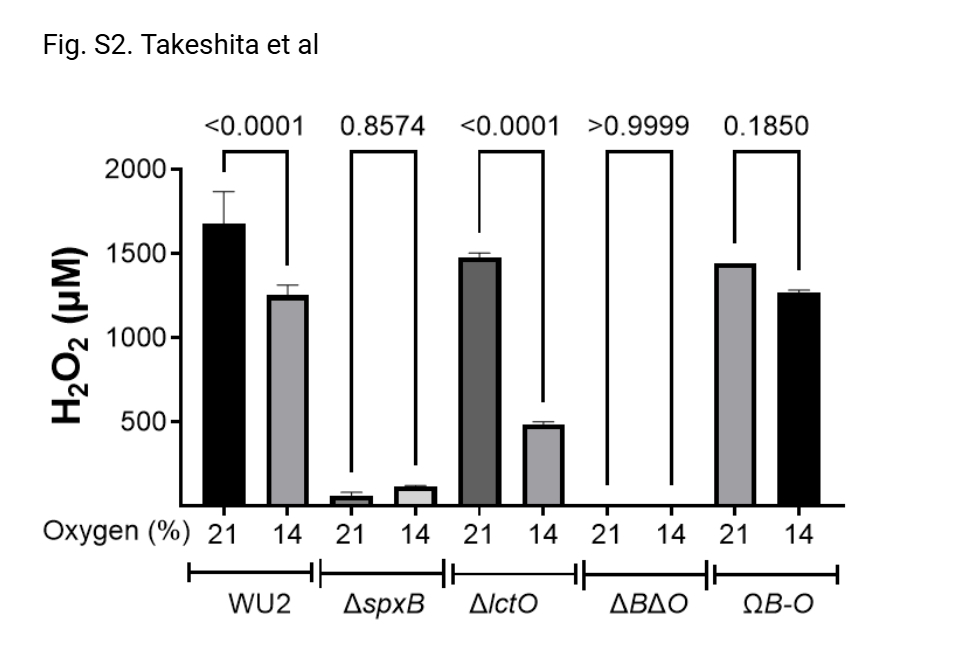

Supplement: Supplement 2 — Figure S2. Hydrogen peroxide production by Streptococcus pneumoniae strain WU2 and mutant derivatives under nasopharyngeal- and lung-mimetic oxygen conditions. Wild-type WU2 and its isogenic mutant derivatives (ΔspxB, ΔlctO, ΔspxBΔlctO, and ΔspxBΩspxB) were inoculated in Todd-Hewitt broth supplemented with 0.5% yeast extract and incubated for 6 h at 37°C under either nasopharyngeal-mimetic conditions (21% O2 + 5% CO2) or lung-mimetic conditions (14% O2 + 5% CO2). Culture supernatants were harvested, filter-sterilized, and extracellular hydrogen peroxide (H2O2) concentration was quantified using the Amplex Red® hydrogen peroxide/peroxidase assay kit. Data are presented as mean ± SE from three independent biological replicates. Statistical comparisons were performed using one-way ANOVA followed by Šidák’s multiple-comparisons test. ns, not significant; *p < 0.05; **p < 0.01; ***p < 0.001; ****p < 0.0001 (exact significance levels are indicated in the figure panels where applicable). [file media-2.jpg]

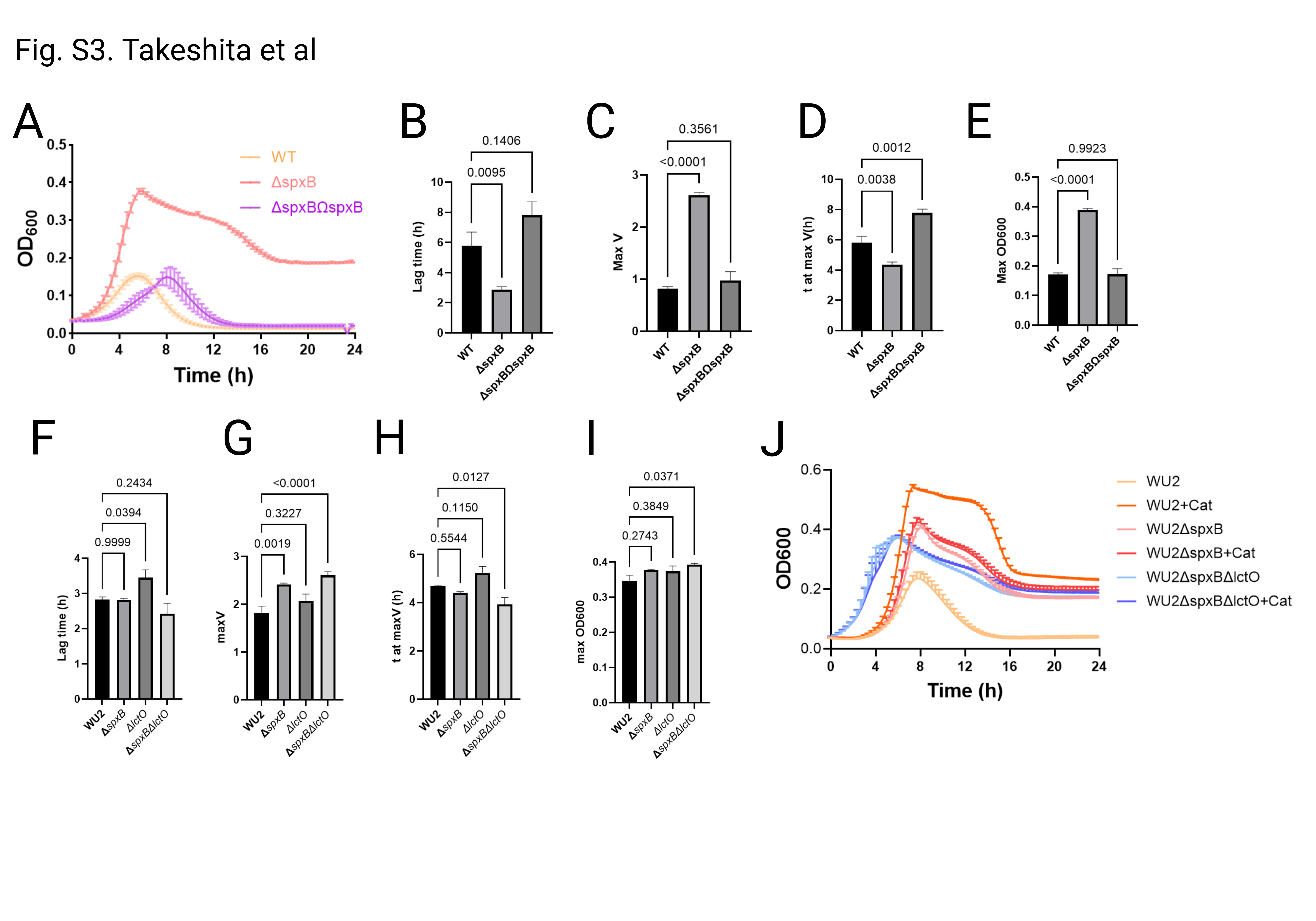

Supplement: Supplement 3 — Figure S3. Growth kinetics of WU2, isogenic mutants, and spxB-complemented strain under varying oxygen conditions, and effects of catalase supplementation. Wild-type WU2, WU2ΔspxB, WU2ΔlctO, WU2ΔspxBΔlctO, and the spxB-complemented strain (WU2ΔspxBΩspxB) were inoculated in Todd-Hewitt broth supplemented with 0.5% yeast extract (THY) and incubated at 37°C under controlled oxygen conditions. Optical density at 600 nm (OD600) was measured every 20 min over 24 h. (A) Growth curves (OD600 vs. time) of ST3 strains under nasopharyngeal-mimetic conditions (21% O2). (B–E) Growth parameters derived from the curves in (A): (B) lag time (h), (C) maximum growth rate (Max V, h−1), (D) time to maximum growth rate (t at Max V, h), and (E) maximum optical density (Max OD600). (F–I) Growth parameters of the ST3 strains under lung-mimetic conditions (14% O2): (F) lag time (h), (G) maximum growth rate (Max V, h−1), (H) time to maximum growth rate (t at Max V, h), and (I) maximum optical density (Max OD600). (J) Growth curves (OD600 vs. time) for wild-type WU2, WU2ΔspxB, and WU2ΔspxBΔlctO cultured under nasopharyngeal conditions (21% O2) in THY, with or without addition of 100 U/mL catalase. Data in all panels represent mean ± SE from at least three independent biological replicates. Statistical comparisons were performed using one-way ANOVA followed by Šidák’s multiple-comparisons test. ns, not significant; exact significance levels are indicated in the figure panels where applicable. [file media-3.jpg]

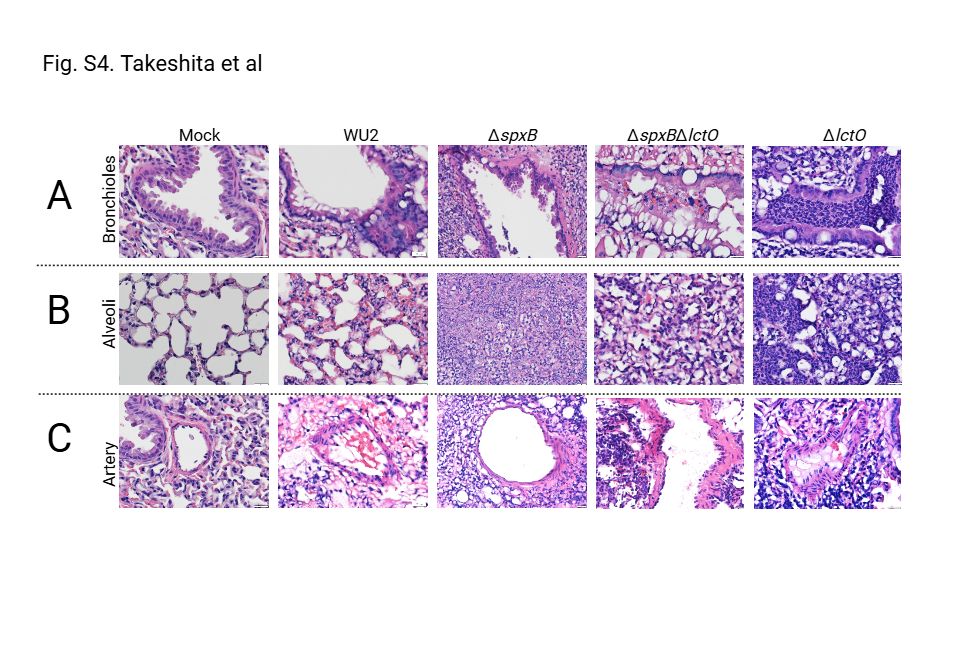

Supplement: Supplement 4 — Fig. S4. Histopathological findings in the lungs of mice infected with serotype 3 Streptococcus pneumoniae. (A–C) Representative hematoxylin and eosin-stained lung sections of the left lungs from mock-infected (PBS) or mice infected with Wild-type WU2, WU2ΔspxB, WU2ΔlctO, or WU2ΔspxBΔlctO at the experimental endpoint. (A) Bronchioles, (B) Alveoli, and (C) Pulmonary arteries/arterioles. Images are representative of n=3–5 mice per group. [file media-4.jpg]
